# Bisphenol A Exposure during Adulthood Alters Expression of Aromatase and 5α-Reductase Isozymes in Rat Prostate

**DOI:** 10.1371/journal.pone.0055905

**Published:** 2013-02-06

**Authors:** Beatriz Castro, Pilar Sánchez, Jesús M. Torres, Ovidiu Preda, Raimundo G. del Moral, Esperanza Ortega

**Affiliations:** 1 Department of Biochemistry and Molecular Biology, School of Medicine, University of Granada, Granada, Spain; 2 Institute of Neurosciences, School of Medicine, University of Granada, Granada, Spain; 3 Department of Pathology, School of Medicine and IBIMER, University of Granada, Spain; Universidad Miguel Hernández de Elche, Spain

## Abstract

The high incidence of prostate cancer (PCa) and benign prostatic hypertrophy (BPH) in elderly men is a cause of increasing public health concern. In recent years, various environmental endocrine disruptors, such as bisphenol A (BPA), have been shown to disrupt sexual organs, including the prostate gland. However, the mechanisms underlying these effects remain unclear. Because androgens and estrogens are important factors in prostate physiopathology, our objective was to examine in rat ventral prostate the effects of adult exposure to BPA on 5α-Reductase isozymes (5α-R types 1, 2, and 3) and aromatase, key enzymes in the biosynthesis of dihydrotestosterone and estradiol, respectively. Adult rats were subcutaneously injected for four days with BPA (25, 50, 300, or 600 µg/Kg/d) dissolved in vehicle. Quantitative RT-PCR, western blot and immunohistochemical analyses showed lower mRNA and protein levels of 5α-R1 and 5α-R2 in BPA-treated groups versus controls but higher mRNA levels of 5α-R3, recently proposed as a biomarker of malignancy. However, BPA treatment augmented mRNA and protein levels of aromatase, whose increase has been described in prostate diseases. BPA-treated rats also evidenced a higher plasma estradiol/testosterone ratio, which is associated with prostate disease. Our results may offer new insights into the role of BPA in the development of prostate disease and may be of great value for studying the prostate disease risk associated with exposure to BPA in adulthood.

## Introduction

Bisphenol A (BPA) is an environmental endocrine disruptor used extensively in the production of polycarbonate plastics and in the epoxy linings of food and beverage cans as well as in dental products [1], [2]. Exposure to BPA is nearly universal; in a recent study, BPA was detected in urine samples from 92.6% of a population in the USA [3]. The widespread exposure of humans to BPA and its potential clinical consequences have recently attracted considerable attention from scientists, government advisors, and the popular press. Moreover, the tolerable daily intake (TDI) of BPA (50 µg/Kg/d) established by the USA Environmental Protection Agency (EPA) called into question after reports of adverse effects below this dose [4].

BPA has been shown to exert endocrine-disrupting effects on reproduction, development, metabolism, and cancer in humans and other species [5], [6]. Recent findings linked exposure to BPA with several male reproductive disorders [7]–[10] and prostate diseases [11]–[13], although the specific mechanisms of action have yet to be elucidated. Most studies have demonstrated that administration of low-doses of BPA during early life, when tissues are especially sensitive to endocrine disrupting chemicals, significantly affect several aspects of prostate development [14], [15]. However, little data have been published on the effects of adult exposure to BPA on the prostate gland. Chitra *et al.* [10] have shown that administration of BPA to adult rat at low dose increases weight of ventral prostate. Other authors have demonstrated that adult exposure to high-dose of BPA can induce expression of cytokeratin 10 (a protein related with squamous metaplasia) in mouse prostatic epithelium [16].

Androgens are essential for the development, maturation, and function of the prostate gland and have also been implicated in benign and malignant prostate disorders, such as benign prostatic hypertrophy (BPH) and prostate cancer (PCa) [17], [18], which are highly common diseases among males in industrialized countries [19]. The most potent androgen in the prostate is 5α-dihydrotestosterone (DHT), obtained from circulating testosterone (T) by the enzyme 5α-Reductase (5α-R, EC 1.3.99.5), which is expressed as three isozymes, types 1, 2, and 3 [20], [21]. 5α-R1 and 5α-R2 isozymes play an important role in prostate disease, and a specific 5α-R2 inhibitor, finasteride, and the dual inhibitor of 5α-R1 and 5α-R2, dutasteride, have been largely used in the treatment of BPH and PCa [22], [23]. Less is known about 5α-R3 isozyme. It has been reported that 5α-R3 may play an important role in protein glycosylation [24]. Mutations of 5α-R3 result in congenital disorders [24], [25] and Kahrizi syndrome [26]. Furthermore, over-expression of 5α-R3 has been reported in different cancer types and it has been proposed as a biomarker of malignancy [27].

Recently published data suggest a role for estrogen in prostate pathogenesis *via* multiple mechanisms [28], including direct genotoxicity, epigenotoxicity, hyperprolactinemia, chronic inflammation and prostatic estrogen receptor-mediated events [29]. It is supported by previous evidence of reduced plasma T levels and elevated plasma estradiol (E2) levels in patients with BPH [30], [31] and of a higher E2/T ratio in aging men at greater risk of prostate disease [28]. However, the variation in serum concentrations of E2 may not accurately reflect intraprostatic levels, because E2 is produced from T by the enzyme aromatase (CYP19A1, EC1.14.14.1), which interestingly is increased in the malignant human prostate [32]. Aromatase expression is known to be modified by BPA in various tissues [33], [34], but this effect has not been explored to date in the adult prostate. Given that BPA is capable of binding to estrogen receptors and androgen receptor in prostate [13] and that T is converted to DHT by 5α-R isozymes, we hypothesized that BPA may also modify 5α-R isozymes.

With this background, the objective of this study was to examine in rat ventral prostate the effects of adult BPA exposure on the expression of aromatase and 5α-R isozymes and explore the possible molecular mechanisms of action of BPA in the development of prostate diseases.

## Materials and Methods

### Animals

Experiments were performed strictly in accordance with recommendations in the Guide for the Care and Use of Laboratory Animals of the National Institutes of Health. Animal care and experimental procedures were approved by the Animal Experimentation Ethics Committee of the University of Granada, Spain (Ref. 412-2012-CEEA).

Adult male Wistar rats weighing 260–280 g were housed in an air-conditioned room with fluorescent lights on from 08:00 to 20:00 and given standard laboratory pellet chow (Panlab rodent chow, Barcelona, Spain). Although the concentration of phytoestrogens in the diet was not evaluated, all animals were exposed to the same phytoestrogen levels because the food intake was equivalent for control and BPA-treated rats. Exposure to environmental endocrine disruptors was minimized by housing the rats in stainless steel cages and using glass bottles with rubber stoppers to supply them with tap water.

### BPA exposure

We evaluated the effect of BPA at the TDI (50 µg/Kg/d) and at higher and lower doses, given that xenoestrogens can cause opposite effects according to the dosage [35]. For this purpose, rats were subcutaneously injected daily for 4 days with 0.2 mL sesame oil containing BPA (Sigma-Aldrich >99% purity) at doses of 25, 50, 300, or 600 µg/Kg/d or with sesame oil alone (controls). Each study group comprised 8 animals.

### Sample processing

At 30 min after the final injection, rats were euthanized by decapitation, and the prostate was removed, frozen in liquid nitrogen, and stored at −80°C until analysis. Blood samples were collected in heparinized tubes. The blood was centrifuged at 2000 rpm for 10 min. The plasma was separated and stored at −20°C until the hormone analysis.

### Hormone Assays

Plasma T concentrations were measured by RIA using a commercial DiaSorin kit (Vercelli, Italy) without modifications; intra- and inter-assay coefficients of variation were 7.6% and 12.0%, respectively, and the sensitivity was 0.05 ng/mL. Plasma E2 concentrations were measured by RIA using a commercial DiaSorin kit (Vercelli, Italy) without modifications; intra- and inter-assay coefficients of variation were 4.8% and 9.5%, respectively, and the sensitivity was 12 pg/mL.

### RNA extraction and reverse transcription

Total RNA was extracted from 50 mg of rat ventral prostate tissue with Trizol reagent (Invitrogen) according to the instructions of the *Sanger Institute*©. RNA samples were then treated with Turbo-DNAse (Ambion) to remove any contamination with genomic DNA. The quantity and purity was determined by using a NanoDrop ND-1000 spectrophotometer (A260/280 ratio), and the integrity was tested by means of denaturing gel electrophoresis. First-strand cDNA was synthesized from 1 µg of total RNA by using MuLV reverse transcriptase (Applied Biosystem). The following agents were added to a final volume of 20 µL reaction: 5 mM MgCl_2_, 1×RT buffer, 1 mM each dNTP, 1 U/µL RNase inhibitor, 2.5 U/µL MuLV reverse transcriptase, 2.5 µM Oligo (dT)_16_, and 1 µg total RNA. Reactions were incubated at 42°C for 15 min, followed by 5 min at 99°C.

### Quantitative Real-Time PCR

Absolute quantification of mRNA of 5α-R1, 5α-R2, 5α-R3, and aromatase in rat prostate tissues was performed by real-time PCR using the Techne Quantica™ Real-time PCR system with SYBR Green PCR Master Mix (Promega). In comparison to relative quantification, this method offers the advantage of giving an absolute copy number for a specific target. The amount of mRNA was expressed as number of mRNA copies per micrograms of total RNA. We amplified tissue samples during real-time PCR in parallel with standard curves, following the method described by Fronhoffs *et al*. [36].

The PCR profile was as follows: denaturation at 94°C for 30 seconds; annealing at 55°C for the Srd5a1 gene, 55°C for the Srd5a2 gene, 50°C for the Srd5a3 gene, 60°C for the Cyp19a1 gene for 30 seconds; and extension at 72°C for 30 seconds. The number of cycles was 40 in all cases. At the end of the amplification phase, a melting curve analysis was carried out on the products formed in order to confirm that a single PCR product was detected by the SYBR Green dye.

Primers for 5α-R1 (Srd5a1 mRNA, Genbank accession n° NM_017070.3), 5α-R2 (Srd5a2 mRNA, Genbank accession n° NM_022711.4), 5α-R3 (Srd5a3 mRNA Genbank accession n° NM_001013990.1) and aromatase (Cyp19a1 mRNA Genbank accession n° NM_017085.2) were designed using Primer 3 software. The primer sequences (5′- 3′) are given in Table 1.

**Table 1:** Primers for PCR amplification of each gene studied.

Primers	Forward primer	Reverse primer
5α-R1	GAGATATTCAGCTGAGACCC	TTAGTATGTGGGCAGCTTGG
5α-R2	ATTTGTGTGGCAGAGAGAGG	TTGATTGACTGCCTGGATGG
5α-R3	TGCCCATCAGTATAAGTGCC	TCACCATAAAGCTCGAACCAG3
Aromatase	TGAGAAGAACGTTCCCTACAG	TCCTCATCTAGATGCAAGGAC

### Immunohistochemical analysis

Expressions of 5α-R1, 5α-R2, and aromatase were determined by immunohistochemistry on formalin-fixed, paraffin-embedded sections of both rat prostate lobules. Tissue sections were treated for 20 min at 98°C in EDTA buffer (1 mM, pH 8) in a PT module (Thermo Fisher, Fremont, CA) for simultaneous dewaxing, hydrating, and antigenic unmasking (retrieval). Immunohistochemical staining was done automatically (Autostainer 480, Thermo Fisher, Fremont, CA) with commercial antibodies against 5α-R1 (goat polyclonal, sc-20658) and 5α-R2 (rabbit polyclonal, sc-20659) (Santa Cruz Biotechnology Inc, Santa Cruz Ca, USA) at 1∶25 dilution and against aromatase (mouse monoclonal clone H4, MCA2077S) (AbD Serotec, Oxford, UK) at 1∶50 dilution. Immunoenzyme peroxidase polymer (anti-goat and Universal; anti-mouse & anti-rabbit) and diaminobenzidine chromogen (Master Diagnóstica, Granada, Spain) were used as detection systems. A dark-brown cytoplasmic and/or nuclear staining was considered positive. Non-specific polyclonal immunoglobulin or IgG isotype was used as negative control for the corresponding antibody. Immunohistochemical expressions of 5α-R1, 5α-R2 and aromatase were semiquantitatively evaluated using a 4-point scale (0, absence; 1, mild; 2, moderate; 3, high) for glandular and/or stromal cell expression.

### Electrophoresis and Western blot analysis

Cytoplasmic and nuclear proteins were extracted from 40 mg of rat ventral prostate tissue with NE-PER extraction reagent (Thermo Scientific), adding protease inhibitor cocktail (Thermo Scientific) according to the manufacturer's protocol. Protein concentration was determined by the dye-binding method of Bradford [37] with BSA as the standard using Bio-Rad protein assay reagent (Bio-Rad Laboratories, Inc, Richmond, CA, USA). Aliquots of samples containing 50 µg of proteins were subjected to 12% sodium dodecyl sulfate-polyacrylamide gel electrophoresis (SDS-PAGE). After electrophoresis, the proteins were transferred from the gel to PVDF blotting membranes in a buffer containing 25 mM Tris, 190 mM glycine, and 20% methanol. Immunoblotting of the membranes was performed after blocking nonspecific binding with 5% nonfat milk and 0.1% Tween 20 in phosphate-buffered saline (T–PBS) pH 7.5 for 2 h. The blots were incubated overnight at 4°C with primary antibodies at a dilution of 1∶500 for 5α-R1, 1∶200 for 5α-R2, 1∶200 for aromatase, 1∶1000 for β-actin, in T-PBS containing 0.5% non-fat dry milk. After several washing with T-PBS, the blots were incubated for 1 h with the appropriate anti Ig G-horseradish peroxidase (HRP) conjugated secondary antibody at a dilution of 1∶5000. After further washings, the blots were visualized using enhanced chemiluminescence detection system according to the supplier's instructions (ECL-Plus, GE Healthcare, USA). The ImageJ program (http://rsb.info.nih.gov/ij/) was used for quantitative analysis of the bands. To account for any differences in loading, target band densitometries were divided by actin densitometries obtained from the same lane. These corrected densitometries were normalized to controls in each experiment.

Antibodies: Goat anti-5α-R1 (Abcam ab110123), rabbit anti-5α-R2 (Santa Cruz sc-20659), mouse anti-aromatase (ABD serotec MCA2077S). A mouse anti β-actin antibody (Thermo Scientific BA3R) was used as loading control. Goat anti-mouse, goat anti-rabbit and donkey anti-goat Ig G HRP conjugated (Santa Cruz) were used as secondary antibodies.

### Statistical Analysis

Data were analyzed by one-way ANOVA. Pairwise comparisons were performed when results proved significant, applying the Tukey method to control for error due to multiple comparisons. P<0.05 was considered significant. All statistical analyses were performed using the STATA Version 10 (Stata Corp. 2007) statistical software.

## Results

### Plasma Testosterone (T), Estradiol (E2), and E2/T ratio

**Figure 1:**
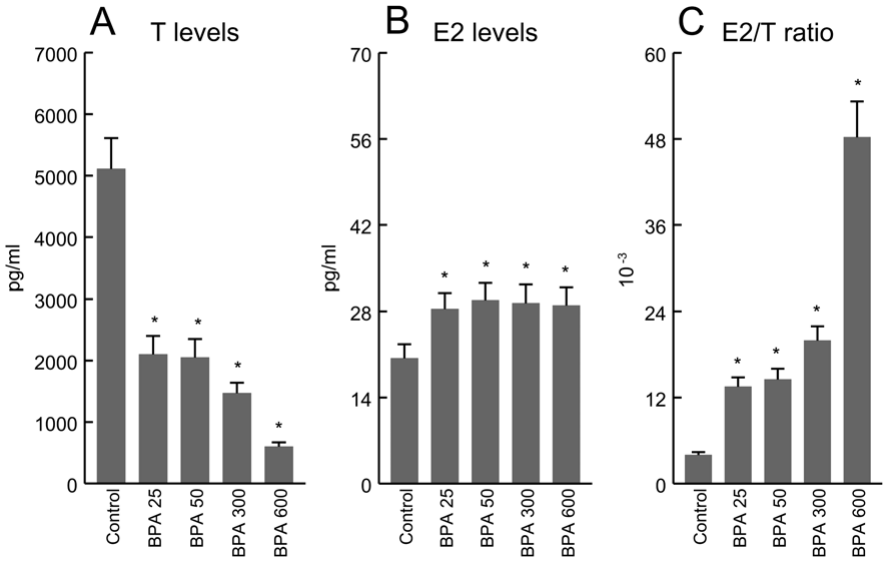
Plasma testosterone (T) concentration (panel A), estradiol (E2) concentration (panel B), and estradiol/testosterone (E2/T) ratio (panel C) in control and BPA-treated rats at doses of 25, 50, 300, or 600 µg/Kg/d for 4 days. *P<0.01 vs. Control animals.

In comparison to values in the controls, plasma T levels were significantly decreased (Fig. 1A) and E2 levels significantly increased in all BPA-treated groups (Fig. 1B), yielding a higher plasma E2/T ratio (Fig. 1C).

### 5α-R1, 5α-R2, 5α-R3 and aromatase mRNA levels

**Figure 2:**
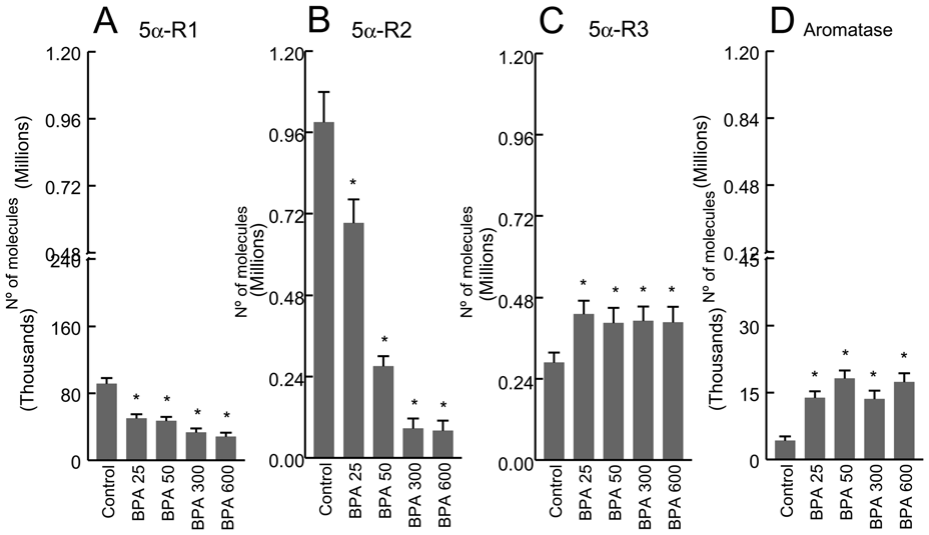
mRNA levels of 5α-Reductase type 1 (5α-R1) (panel A), 5α-Reductase type 2 (5α-R2) (panel B), 5α-Reductase type 3 (5α-R3) (panel C) and aromatase (panel D) in prostate of control and BPA-treated rats at doses of 25, 50, 300, or 600 µg/Kg/d for 4 days. P<0.01 vs. Control animals.

In comparison to the controls, 5α-R1 mRNA levels were significantly decreased in all BPA-treated groups (Fig. 2A), with no significant differences among them. 5α-R2 mRNA levels were significantly decreased in all BPA-treated groups *versus* controls, and the lowest level (around 12-fold vs. controls) was found in the group receiving BPA at doses of 300 and 600 µg/Kg/d (Fig. 2B). In comparison to the controls, 5α-R3 mRNA levels were significantly increased in all BPA-treated groups, with no significant differences among them (Fig. 2C). Likewise, aromatase mRNA levels were significantly increased in all BPA-treated groups *versus* controls, with no significant differences among them (Fig. 2D).

### 5α-R1, 5α-R2, and aromatase expression

#### Immunohistochemistry

**Figure 3:**
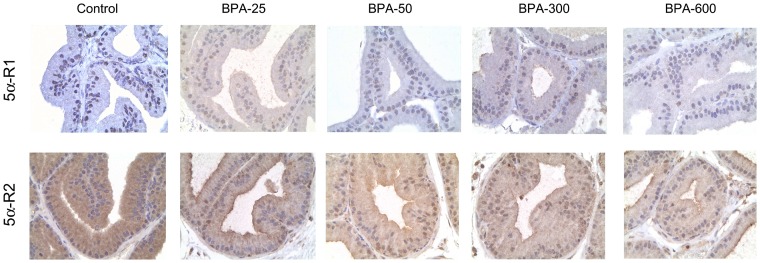
Immunohistochemical staining of 5α-Reductase type 1 (5α-R1) and 5α-Reductase type 2 (5α-R2) in prostate of control and BPA-treated rats at doses of 25, 50, 300, or 600 µg/Kg/d for 4 days. Magnification ×400.

**Figure 4:**
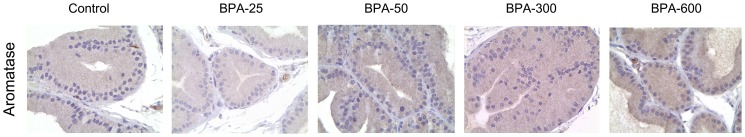
Immunohistochemical staining of aromatase in prostate of control and BPA-treated rats at doses of 25, 50, 300, or 600 µg/Kg/d for 4 days. Magnification ×400.

Microscopic examination of the ventral prostate immunostained slides showed 5α-R1 expression in epithelial cells, mainly in the nucleus (Fig. 3), and 5α-R2 expression in the cytoplasm of both epithelial and stromal cells, with additional nuclear staining in the BPA-treated rats (Fig. 3 and Fig. S1). Expression of both 5α-R1 and 5α-R2 was lower in the BPA-treated groups than in the control group. Unfortunately, there are no commercially available antibodies at present for 5α-R3. Aromatase was expressed mostly in the cytoplasm of the epithelial cells; aromatase expression was higher in BPA-treated rats than in the control group, except at the 25 µg/Kg/d dose (Fig. 4 and Fig. S2).

### Western Blot

**Figure 5:**
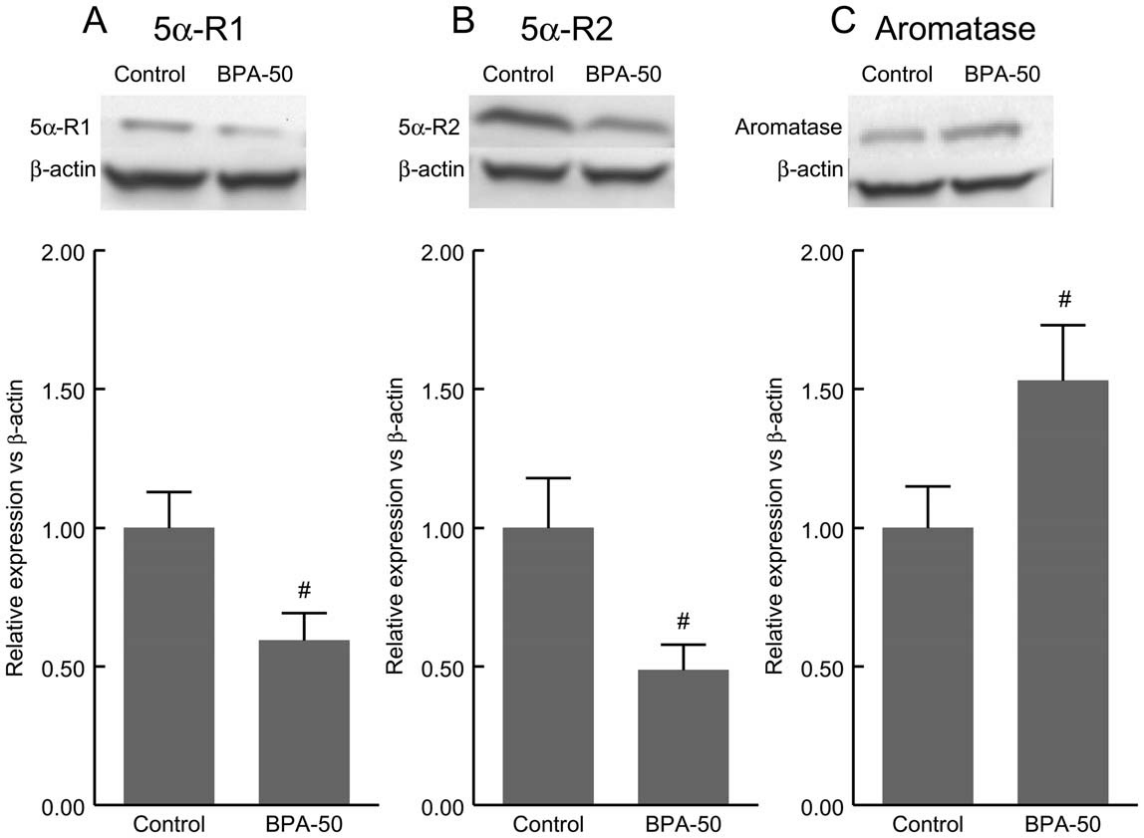
Western blots for the detection of 5α-Reductase type 1 (5α-R1) (panel A), 5α-Reductase type 2 (5α-R2) (panel B) and aromatase (panel C) in prostate of control and BPA-treated rats at tolerable daily intake (TDI) dose of 50 µg/Kg/d for 4 days. Equal loading of protein was determined using an anti β-actin antibody. A representative image of three independent experiments is shown for each blot. Normalization for loading differences was achieved by dividing the densitometry values for individual bands by the densitometry values for β-actin in the same lane. Data represent the mean of three independent experiments. ^#^P<0.05 vs. Control animals.

Figure 5 (panel A) depicts the expression of 5α-R1 isozyme in ventral prostate of control male rats (lane 1) and male rats treated with BPA at the 50 µg/Kg/d dose (TDI) (lane 2). Lower 5α-R1 expression was observed after BPA treatment with respect to control rats.

Figure 5 (panel B) depicts the expression of 5α-R2 isozyme in ventral prostate of control male rats (lane 1) and male rats treated with BPA at the 50 µg/Kg/d dose (TDI) (lane 2). Lower 5α-R2 expression was observed after BPA treatment with respect to control rats.

Figure 5 (panel C) depicts the expression of aromatase in ventral prostate of control male rats (lane 1) and male rats treated with BPA at the 50 µg/Kg/d dose (TDI) (lane 2). Higher aromatase expression was observed after BPA treatment with respect to control rats.

Equal loading of protein in tissue homogenates was also determined by Western blot using an anti-β-actin antibody. All blots were repeated three times and one representative image is shown.

## Discussion

This study contributes the first evidence that adult exposure to BPA influences the expression of 5α-R isozymes and aromatase in the rat prostate. Our experimental results demonstrate that the short-term administration of BPA to adult rats is associated with a decrease in mRNA and protein levels of both 5α-R1 and 5α-R2 isozymes. Within the prostate 5α-R1 and 5α-R2 are positively regulated by T [38], [39], consistent with the present results. BPA may down regulate these isozymes by reducing circulating T levels through decreasing luteinizing hormone (LH) secretion; or interfering with the LH receptor [40], or inhibiting T biosynthetic enzymes [41]. BPA may also exert these effects through the androgen receptor, although reports have been controversial [42].

The localization of 5α-R2 in this study was mainly in the cytoplasm, in agreement with Span *et al.* [43], although an additional nuclear staining was observed in BPA-treated rats. Interestingly, Thomas *et al.* [44] observed that 5α-R2 was predominantly cytoplasmic in recurrent cancer but some nuclear staining was also present. However, the functional significance of the nuclear localization of 5α-R2 remains still unclear.

Our BPA-treated rats showed increased mRNA levels of 5α-R3 (a suggested malignancy biomarker) in association with decreased plasma T levels. Li *et al.* [38] found a transition from positive to negative regulation of 5α-R3 by androgens in the development and progression of PCa. Although our results point to this line, further studies e.g. longer BPA exposure, are required to link the data at molecular levels after BPA exposure with prostate pathologies.

There is adequate evidence to relate androgens to prostate disease, but other factors may also be implicated, given that the prevalence of prostate pathologies increases with age when circulating T levels decline. Estrogens may also play a critical role in predisposing for or causing prostate disease [45]. Numerous epidemiological studies have reported a relationship between elevated circulating estrogen levels or E2/T ratio and prostate disease, including PCa [28]. In the present study, an increase in E2 and decrease in T, i.e., a higher E2/T ratio, were induced by the administration of BPA.

If elevated intraprostatic E2 levels are involved in PCa [46], aromatase may play an important role in the development of this disease. The present results demonstrate that BPA administration increases aromatase mRNA and protein levels in the prostate of adult rats. However, there was a lower increase in protein than in mRNA levels of this enzyme at a dose of 25 µg/Kg/d. There may possibly be some post-transcriptional defense mechanism to avoid aromatase gene overexpression at low doses of BPA.

To our knowledge, this is the first report on the effects of BPA on aromatase in adult rat prostate. Arase *et al.* [33] demonstrated that fetal exposure to low-dose BPA increases *in situ* E2 and aromatase mRNA and enzymatic activity in the mouse urogenital sinus from which the prostate develops. However, *in vitro* studies in other cellular types found decreased aromatase levels after BPA administration [47], [48]. Differences between the adult and the developing prostate, animal, tissue, endocrine system, and in vitro versus in vivo studies should be kept in mind.

## Conclusions

The present study contributes the first evidence that adult exposure to BPA modifies in the rat prostate gland the expression of 5α-R isozymes and aromatase, key enzymes in the prostatic physiopathology. These effects were observed over the short-term and at levels at or below the TDI for this compound. Our study opens up a new line of investigation on the risk of prostate disease associated with exposure to BPA in adulthood.

## Supporting Information

Figure S1 **Immunohistochemical staining of 5α-Reductase type 1 (5α-R1) and 5α-Reductase type 2 (5α-R2) in prostate of control and BPA-treated rats at doses of 25, 50, 300, or 600 µg/Kg/d for 4 days. Magnification ×200.**
(TIF)

Figure S2 **Immunohistochemical staining of aromatase in prostate of control and BPA-treated rats at doses of 25, 50, 300, or 600 µg/Kg/d for 4 days. Magnification ×200.**
(TIF)
