# Supplementary figures and images for: Bisphenol A Exposure during Adulthood Alters Expression of Aromatase and 5α-Reductase Isozymes in Rat Prostate
Source: PLoS One. 2013 Feb 6;8(2):e55905. doi: 10.1371/journal.pone.0055905 (PMC3566099; doi:10.1371/journal.pone.0055905)

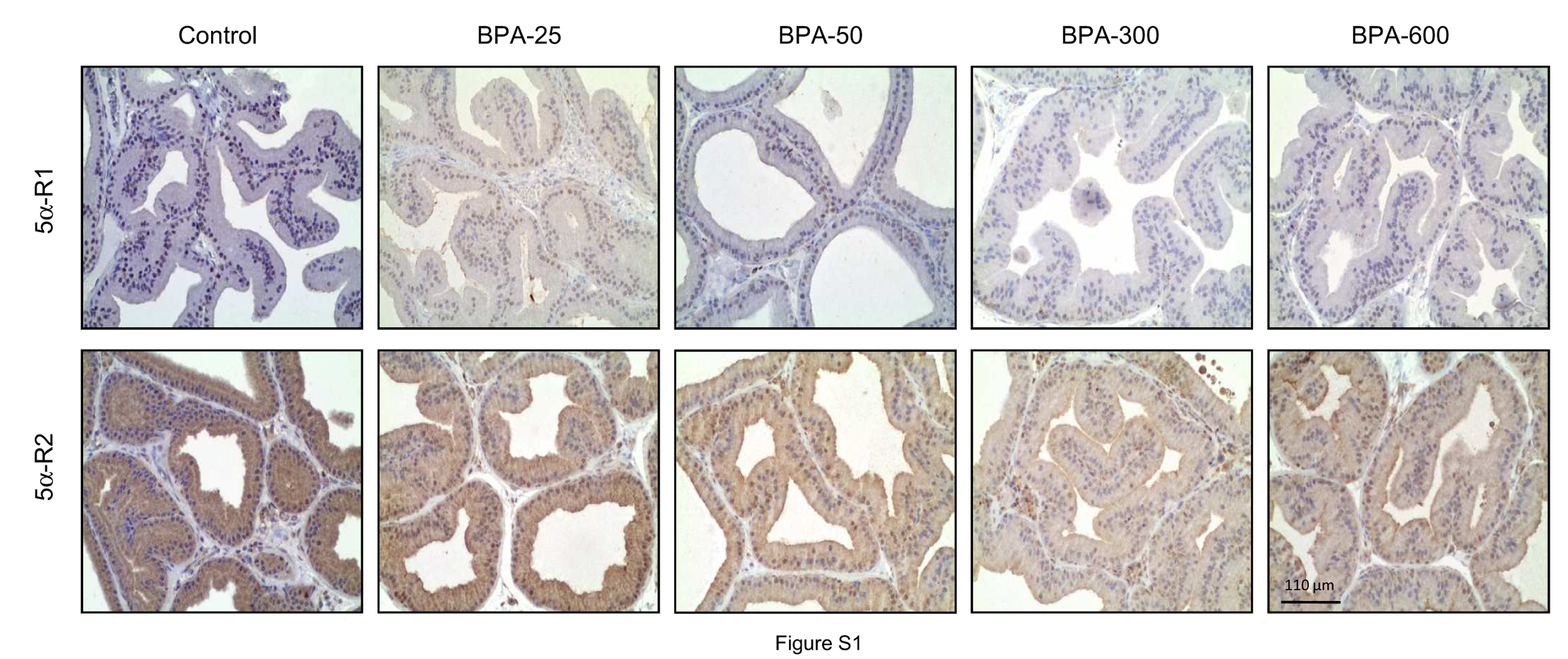

Supplement: Figure S1 — Immunohistochemical staining of 5α-Reductase type 1 (5α-R1) and 5α-Reductase type 2 (5α-R2) in prostate of control and BPA-treated rats at doses of 25, 50, 300, or 600 µg/Kg/d for 4 days. Magnification ×200. (TIF) [file pone.0055905.s001.tif]

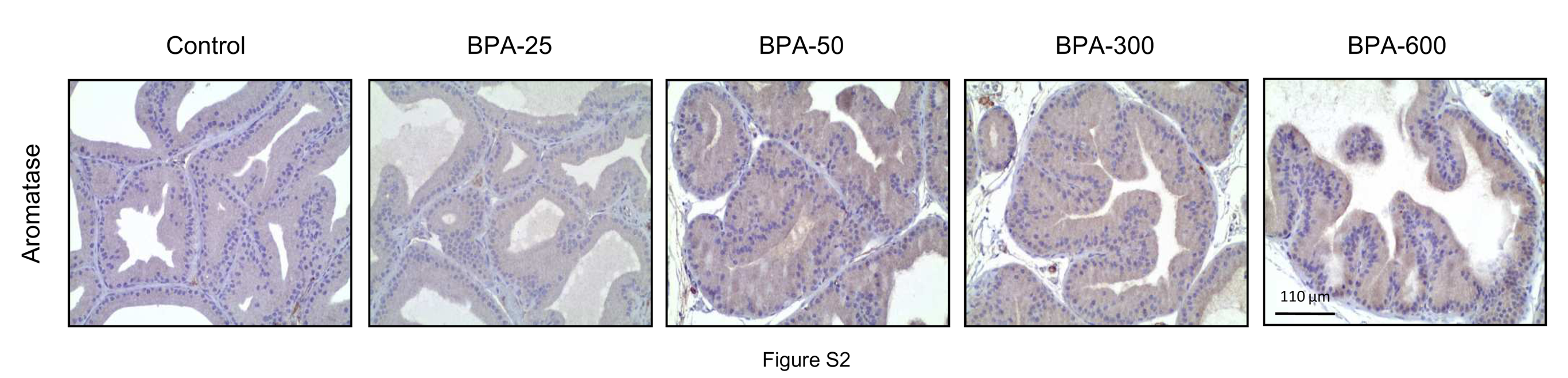

Supplement: Figure S2 — Immunohistochemical staining of aromatase in prostate of control and BPA-treated rats at doses of 25, 50, 300, or 600 µg/Kg/d for 4 days. Magnification ×200. (TIF) [file pone.0055905.s002.tif]
